# Tumor-specific enhanced NIR-II photoacoustic imaging via photothermal and low-pH coactivated AuNR@PNIPAM-VAA nanogel

**DOI:** 10.1186/s12951-024-02617-y

**Published:** 2024-06-10

**Authors:** Xiaodong Sun, Yujie Li, Xiaowan Liu, Dandan Cui, Yujiao Shi, Guojia Huang

**Affiliations:** 1 MOE Key Laboratory of Laser Life Science & Institute of Laser Life Science, College of Biophotonics, South China Normal University, Guangzhou, 510631 China; 2 Guangdong Provincial Key Laboratory of Laser Life Science, College of Biophotonics, South China Normal University, Guangzhou, 510631 China; 3 Reproductive Medicine Research Center, The Sixth Affiliated Hospital, Sun Yat-Sen University, Guangzhou, 510655 China; 4 Biomedical Innovation Center, The Sixth Affiliated Hospital, Sun Yat-Sen University, Guangzhou, 510655 China; 5 Medical Research Institute, Guangdong Provincial People’s Hospital (Guangdong Academy of Medical Sciences), Southern Medical University, Guangzhou, 510080 China

**Keywords:** Photoacoustic imaging; phase transition, NIR-II, Nanogel, Tumor detection

## Abstract

**Background:**

Properly designed second near-infrared (NIR-II) nanoplatform that is responsive tumor microenvironment can intelligently distinguish between normal and cancerous tissues to achieve better targeting efficiency. Conventional photoacoustic nanoprobes are always “on”, and tumor microenvironment-responsive nanoprobe can minimize the influence of endogenous chromophore background signals. Therefore, the development of nanoprobe that can respond to internal tumor microenvironment and external stimulus shows great application potential for the photoacoustic diagnosis of tumor.

**Results:**

In this work, a low-pH-triggered thermal-responsive volume phase transition nanogel gold nanorod@poly(n-isopropylacrylamide)-vinyl acetic acid (AuNR@PNIPAM-VAA) was constructed for photoacoustic detection of tumor. Via an external near-infrared photothermal switch, the absorption of AuNR@PNIPAM-VAA nanogel in the tumor microenvironment can be dynamically regulated, so that AuNR@PNIPAM-VAA nanogel produces switchable photoacoustic signals in the NIR-II window for tumor-specific enhanced photoacoustic imaging. In vitro results show that at pH 5.8, the absorption and photoacoustic signal amplitude of AuNR@PNIPAM-VAA nanogel in NIR-II increases up obviously after photothermal modulating, while they remain slightly change at pH 7.4. Quantitative calculation presents that photoacoustic signal amplitude of AuNR@PNIPAM-VAA nanogel at 1064 nm has ~ 1.6 folds enhancement as temperature increases from 37.5 °C to 45 °C in simulative tumor microenvironment. In vivo results show that the prepared AuNR@PNIPAM-VAA nanogel can achieve enhanced NIR-II photoacoustic imaging for selective tumor detection through dynamically responding to thermal field, which can be precisely controlled by external light.

**Conclusions:**

This work will offer a viable strategy for the tumor-specific photoacoustic imaging using NIR light to regulate the thermal field and target the low pH tumor microenvironment, which is expected to realize accurate and dynamic monitoring of tumor diagnosis and treatment.

## Introduction

The tumor microenvironment (TME) is a complex network of cancer-associated cells and non-cellular components [1]. The characteristics of the TME, such as redox state, reactive oxygen species, low pH, tumor-associated receptors, etc. provide promising triggers for the stimulus response feature used in cancer diagnosis and therapy [2, 3]. In particular, compared with the limited concentration of tumor-associated receptors, acidic tumor interstitial and local high temperature are more likely targets for stimulus response. On the one hand, under normal physiological conditions, the pH value of blood and tissue is strictly controlled at about 7.4 [4]. However, in diseased tissues such as tumors, the TME is acidic, with a local pH of approximately 5.5 to 7.0, due to glycolytic cancer cell metabolism, hypoxia, and hypoperfusion [5, 6]. Generally, acidic pH has a pleiotropic effect on the proliferation, migration, invasion, metastasis, therapeutic response of cancer cells, as well as the function of immune cells, vascular cells and other stromal cells, and further promotes the malignancy and development of tumors [7–10]. Therefore, the diagnosis and treatment method that responds to the characteristics of TME is very important for the diagnosis and treatment of tumor.

Imaging forms an essential part of cancer clinical protocols and is able to furnish morphological, structural, metabolic and functional information [11–13]. Photoacoustic imaging (PAI), as a new non-invasive medical imaging method, combines pure optical imaging with ultrasonic imaging, and successfully circumvented the “soft limit” that the imaging depth of traditional optical imaging can only reach 1 mm in principle [14, 15, 16]. Generally, subjected to different methods of optical illumination and acoustic detection, PAI systems can be grouped into computed tomography (or tomography), mesoscopy and microscopy (PAM) [17–21]. PAI has major potential for clinical applications in the future [22, 23]. In particular, the tissue penetration depth of PAI are greatly improved compared to fluorescence imaging techniques [24]. Compared to NIR-I in the range of 700–900 nm, NIR-II (1000–1700 nm) PAI possesses higher sensitivity and penetration depth [25, 26]. However, exogenous photoacoustic probes with higher efficacy and deeper tissue penetration in the NIR-II window have not been fully developed. Compared with conventional photoacoustic nanoprobes where photoacoustic signals are always “on”, TME-responsive nanoprobes can minimize the influence of endogenous chromophore background signals [27, 28]. Furthermore, it is difficult for a single endogenous or external stimulus to achieve an ideal effect. The use of stimuli-response nanoprobes to achieve collaborative response of TME features will further improve PAI performance [29–32].

In our previous work, our research group has carried out a series of work on the diagnosis of diseases by PAI technology [33–36], as well as the development of stimuli-responsive photoacoustic nanoplatforms in NIR-II region [37–40]. A high-performing thermal-responsive polyethylene glycol-coated tungsten-doped vanadium dioxide (W-VO_2_@PEG) nanoprobe with strong and switchable optical absorption in the NIR-II biowindow near humanbody temperature, has been investigated to achieve deep and contrast-enhanced PAI [37]. Furtherly, W-VO_2_@PEG was applied to realize quantitative 3D temperature rendering of deep tumors to promote precise cancer photothermal therapy [38]. Based on the glutathione characteristics in the TME, a NIR-II ultrahigh-sensitive and tumor-specific photoacoustography technique was developed by introducing a photosensitive self-synthesized photosensitive silver bromide modified with poly(lactic-co-glycolic acid) (AgBr@PLGA) nanocrystals, which are fixed graphic by optical writing and redox-responsive [39]. An intelligent all-in-one theranostic nanoprobe (PEG/αCD25-Cy7/TMZ) with glutathione sensitivity was developed to precisely deliver TMZ for local chemotherapy and in situ trace Treg cells by photoacoustic-fluorescence imaging [40]. Therefore, the development of nanoprobe that can respond to internal TME or external stimulus shows great application potential in the PAI diagnosis of tumor, which impelled us to further investigate the new intelligent nanoprobe with both endogenous low pH and exogenous thermal-response characteristics for high-precision diagnosis and treatment of tumor taking the special advantages of PAI.

In this work, a low-pH-triggered thermal-sensitive volume phase transition nanoprobe gold nanorod@poly(n-isopropylacrylamide)-vinyl acetic acid (AuNR@PNIPAM-VAA) was constructed to dynamically regulate the absorption of NIR-II light in the TME via an external near-infrared optical switch, thus output enhanced and switchable photoacoustic signals in the NIR-II window for tumor-specific high-contrast PAI. pH responsive functional group was introduced by copolymerizing n-isopropylacrylamide with vinylacetic acid (VAA) monomers, and adjusted the monomer ratio to further imbue PNIPAM-VAA shell with the pH-dependence required for thermal-sensitive phase transitions to target acid TME. When the temperature exceeded the volume phase transition temperature (VPTT), the developed AuNR@PNIPAM-VAA nanogel underwent a solgel phase transition in TME and further resulted in enhanced NIR-II light absorption due to the change of nanogel’ refractive index. The thermal-responsive and pH-dependent ability of the AuNR@PNIPAM-VAA nanogel was investigated under different conditions. Finally, in vivo experiments were carried out to examine the capacity of AuNR@PNIPAM-VAA nanogel to specifically detect tumor by PAI. The results showed that the prepared AuNR@PNIPAM-VAA nanogel can achieve enhanced contrast PAI for selective tumor detection through dynamically responding to thermal, which was controlled by external light.

## Materials and methods

### Materials

Cetyltrimethylammonium bromide (CTAB), chloroauric acid, sodium borohydride, vinyl acetic acid (VAA), ascorbic acid, hydrochloric acid (HCl), N, N ‘-methylene bisacrylamide (MBA), n-isopropylacrylamide (NIPAM), 2,2’-Azobis(2-methylpropionamidine) dihydrochloride, odium oleate and silver nitrate were purchased from Shanghai Aladdin Biochemical Technology Co., Ltd (Shanghai, China). Phosphate buffered saline (PBS) were received from Gibco (Grand Island, USA). All reagents were used directly with no further treatment. The water was purified using a Milli-Q water purification process (Millipore, Bedford, MA) with a resistivity at 18.2 MΩ·cm.

### Synthesis of AuNR@PNIPAM-VAA

AuNR was synthesized by gold seed growth method as previous literature [41]. (1) The gold seed solution is prepared as follows: 5 mL of 0.2 M CTAB aqueous solution and 5 mL of 0.5 mM chloroauric acid aqueous solution were added into 20 mL scintillation bottle, stirring for 10 min. 600 µL of 0.01 M sodium borohydride solution were added, stirring for 2 min and aged at room temperature for 15 min. (2) Configuration of growth solution: 1.8 g of CTAB and 0.245 g of sodium oleate were dissolved in 50 mL water, then 480 µL of 40 mM silver nitrate aqueous solution were added. The mixture was kept at room temperature for 15 min, and then 50 mL of 1 mM chlorauric acid solution was added and continued stirring for 90 min. The solution changed from yellow to colorless, 3.6 mL,1 M of HCl solution was added to adjust the pH, and the solution was slowly stirred at 400 rpm for 15 min. Then, 400 µL of 40 mM ascorbic acid solution was added under intense agitation, and continued to stir vigorously for 30 s. Finally, 160 µL of seed solution was injected into the growth solution, and the mixture was vigorously stirred for 30 s and kept at room temperature for 12 h. The obtained AuNR was centrifuged twice at 8000 rpm for 20 min each time, the supernatant was removed and dispersed in deionized water.

Fabrication of AuNR@PNIPAM-VAA. 100 mL of AuNR aqueous solution was heated to 70 ℃ and 600 µL of VAA was added to stir for 1 h. After cooling, the AuNR solution was centrifuged at 7500 rpm for 20 min, the supernatant was removed, and the obtained AuNR solution was re-dispersed in 10 mL of deionized water. Above solution was added to a 50 mL schlenk reaction bottle, then 40 µL of VAA, 0.1 g of NIPAM and 0.014 g of MBA were added under magnetic agitation. The solution was heated to 70 ℃ under nitrogen for 15 min, and then 400 µL of 25 mM 2, 2 ‘-Azobis(2-methylpropionamidine) dihydrochloride was added to initiate polymerization for 12 h. The solution was cooled to room temperature. The obtained nanogels were placed in deionized water for dialysis for two days, centrifuged at 7500 rpm for 20 min, and the precipitated AuNR@PNIPAM-VAA composite nanogel were re-dispersed in equal volume of water. Finally, the dry nanogel was obtained after freeze-drying for 48 h and sealed for storage.

### Main instruments

High-resolution transmission electron microscope (HR-TEM) (FEITalos F200X), Transmission electron microscope (TEM) (JEM-1400 PLUS, Japan Electronics), UV-Visible Spectrophotometer (UV-2600, Shimadzu, Japan), Nano-particle size - Zeta potential analyzer (Zetasizer Nano-ZS, Malvern instruments, UK), Photothermal Imager (Fotric 326pro, USA), pH meter (Sartorius, Germany), etc. were applied.

A PAM system was used to evaluate the PAI performance of AuNR@PNIPAM-VAA. A pulsed laser (DS20HE-1064D/R, PHOTONICS) was selected as the excitation light source for PAM. The laser repetition rate was 5 kHz, the wavelength was 1064 nm, and the pulse width was 11 ns. During the experiment, the photoacoustic signals were detected by a single array detector (center frequency,10 MHz; light transmission aperture, 6 mm; bandwidth, 95%; focal length, 15.3 mm). The detected photoacoustic signal passes through the filter (BLP-21.4+, Mini Circuits, New York, USA), is amplified by the amplifier (LNA-650, RF Bay Inc, Maryland, USA), and then transmitted by the data transmission line to the acquisition card for collection. The laser energy density is controlled below 20 mJ/cm^2^.

### Establishment of tumor model

All experiments were approved by the Animal Research Ethics Committee of South China Normal University and were performed by relevant guidelines and regulations. Female BALB/c nude mice (4–5 weeks) were purchased from Animal Center of Southern Medical University. 4T1 cells (1 × 10^6^ cells suspended in 15 µL PBS) were injected subcutaneously into the back of nude mice, and the mice were fed in SPF environment. Tumor volume was calculated as $\text{V}=l{\text{w}}^{2}/2$ in which *l* and *w* indicate the length and width of the tumor, respectively.

### Photothermal imaging

In vitro photothermal performance of AuNR@PNIPAM-VAA nanogel. The temperature rise curves of AuNR@PNIPAM-VAA solution with a mass concentration of 1.5 mg/mL at pH of 7.4 and 5.8 were investigated under the irradiation of 980 nm continuous wave (CW) laser with the same optical power density of 0.75 W/cm^2^. In addition, three groups of AuNR@PNIPAM-VAA aqueous solutions (200 µL, 1 mg/mL) were placed in a centrifugal tube and irradiated for 20 min with optical power densities of 0.25 W/cm^2^, 0.5 W/cm^2^ and 0.75 W/cm^2^, respectively. The temperature change of sample solution was recorded by infrared imager.

Photothermal stability test of AuNR@PNIPAM-VAA nanogel. AuNR@PNIPAM-VAA solution (200 µL, 1 mg/mL) was placed in a centrifugal tube and irradiated with an optical power density of 0.75 W/cm^2^ for 10 min. Then the light source was removed and the AuNR@PNIPAM-VAA solution was cooled naturally for 10 min. This process was repeated six times and the temperature changes were recorded.

In vivo photothermal effects of AuNR@PNIPAM-VAA nanogel. AuNR@PNIPAM-VAA buffer solution (100 µL, 1 mg/mL) at pH of 7.4 and 5.8 were injected subcutaneously into the same tissue site (right back) of two female Bab/c nude mice, respectively. The exposure light source was 980 nm CW laser with an optical power density of 0.5 W/cm^2^. The region of interest was irradiated with a spot of 0.3 cm^2^ for 10 min, and the temperature change of the tissue in the region was recorded.

### Photoacoustic imaging

In vitro photoacoustic imaging of AuNR@PNIPAM-VAA nanogel at different pH values was performed. To confirm the response of thermal and acidity, four polyethylene transparent tubes (diameter: 1 mm, wall thickness: 0.1 mm) were filled with 1 mg/mL of AuNR@PNIPAM-VAA nanogel at pH of 7.4, 6.5, 6.0 and 5.8, respectively and subsequently immersed into a temperature-controllable water bath to further investigated by PAM under 1064 nm at different temperature. To examine the optical switch control ability, the scanning samples were three polyethylene transparent tubes, two of which were injected with AuNR@PNIPAM-VAA (1 mg/mL) buffer at pH 7.4 and pH 5.8 respectively, and irradiated with 980 nm CW laser, while the third one was filled with AuNR@PNIPAM-VAA buffer at pH 5.8 serving as a control group without laser irradiation.

For in vivo PAI, normal mice were injected with 100 µL of AuNR@PNIPAM-VAA aqueous solution (1 mg/mL) with pH 7.4 and 5.8 in the back, respectively. 4T1 tumor-bearing mice were injected with 100 µL of AuNR@PNIPAM-VAA aqueous solution (1 mg/mL). After different periods of irradiation with a 980 nm CW laser (0.5 W/cm^2^), the PAM system was used to perform x-z spot scanning of normal tissue and tumor sections injected with the nanogel. The structural information is provided by ultrasound image. In order to ensure a good coupling between photoacoustic and ultrasonic imaging, the handheld ultrasonic probe is used for ultrasonic imaging immediately at the completion of PAI according to the target region. The direction of the ultrasonic imaging probe and the distance between the sample are consistent with that of the PAI to keep the imaging area size consistent.

### In vivo toxicity evaluation

To evaluate in vivo toxicity of AuNR@PNIPAM-VAA, pathological structure of main organs (heart, liver, spleen, lung and kidney), biochemical and hematological analysis were performed. After intravenous injection of PBS, AuNR@PNIPAM-VAA (100 µL, 1.5 mg/mL), the main organs were harvested, fixed in 10% buffered formalin, processed routinely, stained with hematoxylin and eosin (H&E), and examined by optical microscopy. The serum biochemical and hematological parameters such as white blood cell (WBC), red blood cell (RBC), hemoglobin (HGB), hematocrit test (HCT), mean erythrocyte hemoglobin (MCH), mean erythrocyte hemoglobin concentration (MCHC), platelet (PLT), mean platelet volume (MPV) were investigated systematically.

## Results and discussion

### Preparation of the thermal-pH dual responsive AuNR@PNIPAM-VAA nanogel

Poly(n-isopropylacrylamide) (PNIPAM) is the common thermal-sensitive hydrogel, and its lower critical solution temperature can range from 30 to 35 °C depending on the precise solvent and chain modifications, which is below the physiological temperature of humans and most animals [42, 43]. In this work, VAA monomer was introduced into PNIPAM chain to make the hydrogel with pH-responsive function, which can be adjusted the monomer ratio to further imbue PNIPAM-VAA shell with the pH-dependence required for thermal-sensitive phase transitions in the TME. On the one hand, VPTT of PNIPAM-VAA nanogel in acidic TME (pH = 5.8), which was close to the hyperthermia temperature (45℃) and much lower than that under normal physiological conditions (pH = 7.4), is the most ideal for tumor specific contrast imaging. On the other hand, the absorber with good optical absorption and photothermal conversion efficiency in NIR-II must be taken to consider.

The absorption peak wavelength of nanoparticle depends on its composition, size, shape, orientation and local dielectric environment. The localized surface plasmon resonance spectral shift (Δλ) of AuNR in response to changes in refractive index is approximately described as [44]: ${\Delta }{\uplambda } \approx m({n}_{\text{a}\text{d}\text{s}\text{o}\text{r}\text{b}\text{a}\text{t}\text{e}}-{n}_{\text{m}\text{e}\text{d}\text{i}\text{u}\text{m}})(1-{\text{e}}^{-2d/l} )$, where *m* is the sensitivity factor (in nm per refractive index unit (RIU)), ${n}_{\text{a}\text{d}\text{s}\text{o}\text{r}\text{b}\text{a}\text{t}\text{e}}$ and ${n}_{\text{m}\text{e}\text{d}\text{i}\text{u}\text{m}}$ are the refractive indices (in RIU) of the adsorbate and medium surrounding the AuNR, respectively, *d* is the effective thickness of the adsorbate layer (in nm), and *l* is the electromagnetic field decay length (in nm). Localized surface plasmon resonance shifts are maximized by optimizing the nanoparticle characteristics, *m* and *l*, as well as the Δ*n* due to molecular adsorption. Therefore, we designed a core-shell phase transition nanoprobe with PNIPAM-VAA coated gold nanorods (AuNR@PNIPAM-VAA). The plasmon absorption of AuNR varies with the refractive index of the surrounding environment, when PNIPAM-VAA hydrogel undergoes a thermal-induced sol-gel phase transition near VPTT. As a result, the AuNR exhibits enhanced absorption in NIR-II region, and further generated enhanced photoacoustic signals.

**Fig. 1:**
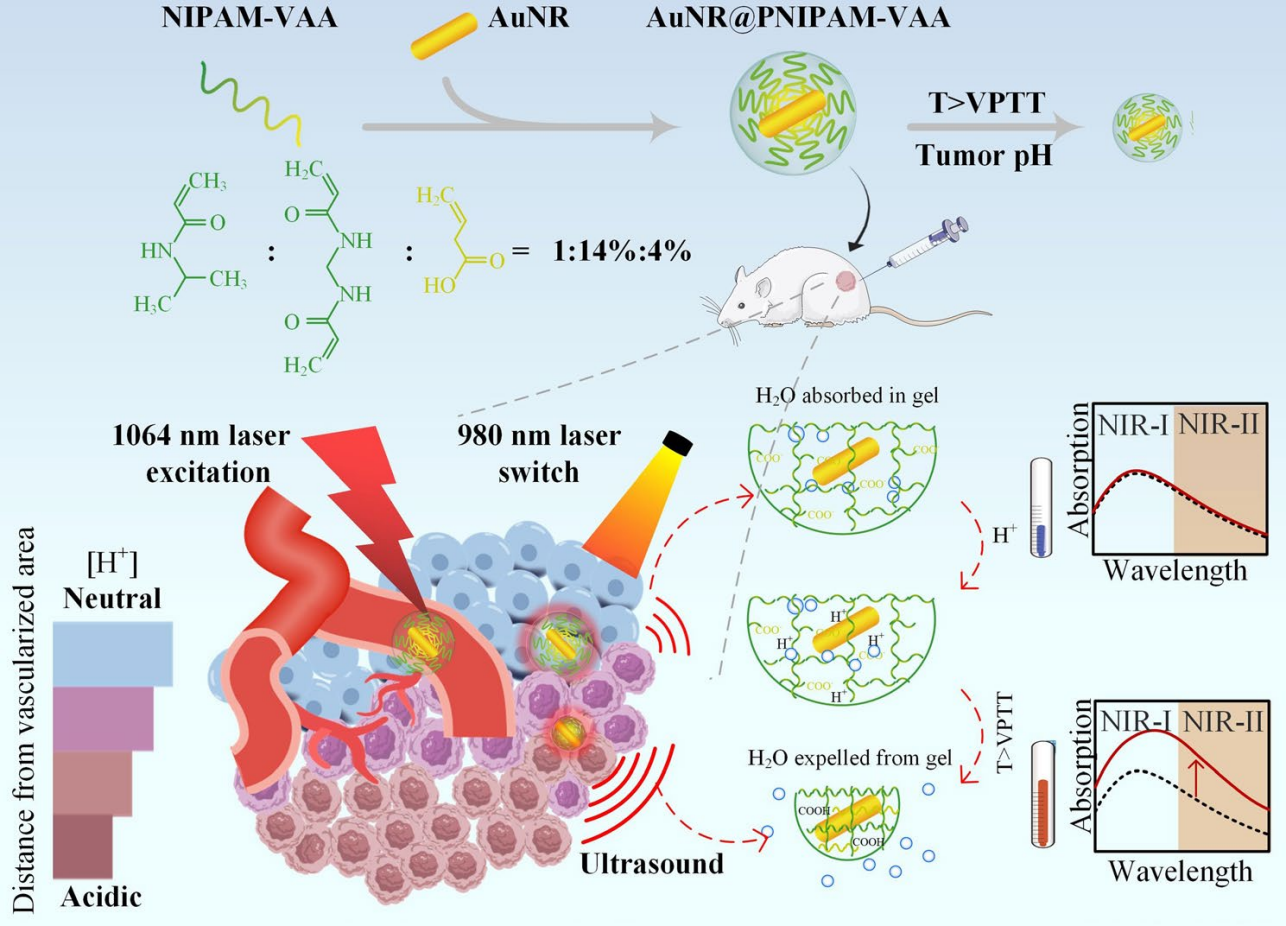
Schematic diagram of the thermal-responsive volume phase transition AuNR@PNIPAM-VAA nanogel triggered by low pH in TME for tumor-specific high contrast PAI.

As shown in Fig. 1, AuNR@PNIPAM-VAA nanogel in the TME was irradiated by 980 nm CW laser, generated heat through photothermal effect and transferred it to the PNIPAM-VAA shell to increase its surrounding temperature. In the TME, when the temperature of the PNIPAM-VAA shell was higher than VTPP, the AuNR@PNIPAM-VAA nanogel underwent a “swelling-shrinkage” volume phase transition, and the refractive index around AuNR increased sharply and was close to that of the dehydrated polymer network (*n* = 1.46) [45]. The longitudinal plasmonic resonance of the probe core-shell nanogel is red-shifted, and the light absorption of NIR-II is sharply enhanced, which makes the photoacoustic signal generated in the NIR-II window continuously enhanced. On the contrary, in normal tissues, PNIPAM-VAA shell was still in a state of swelling as the temperature of PNIPAM-VAA shell was lower than VTPP. The refractive index of PNIPAM-VAA shell remained unchanged, close to that of water (*n* = 1.33), and the photoacoustic signal of AuNR@PNIPAM-VAA nanogel keep the same. In conclusion, low pH in the TME could trigger the thermal-sensitive volume phase transition of AuNR@PNIPAM-VAA nanogel, resulting in enhanced photoacoustic signal output in the NIR-II window.

### Characterization of AuNR@PNIPAM-VAA nanogel

**Fig. 2:**
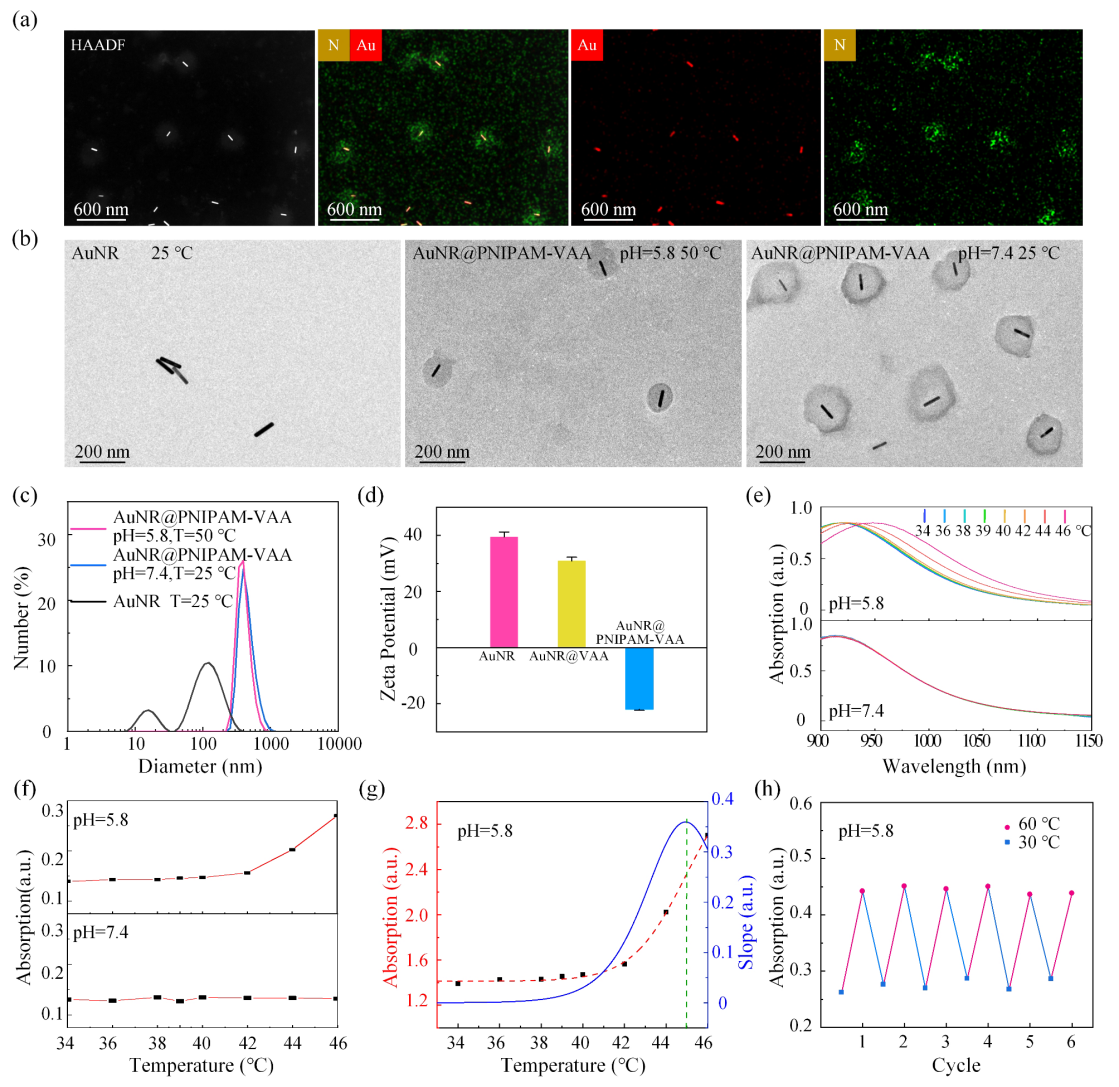
Characterization of AuNR@PNIPAM-VAA nanogel. (**a**) HAADF and element mapping images; (**b**) TEM image of the thermal-response size and morphology; (**c**) DLS analysis and (**d**) Zeta potential of AuNR, AuNR@PNIPAM-VAA ; (**e**) Absorption spectra and (**f**) quantitative absorption intensity at 1064 nm; (**g**) Derivative absorption intensity of AuNR@PNIPAM nanogel with temperature at pH 5.8; (**h**) Reversibility of light absorption at 1064 nm during 30 °C to 60 °C heating and cooling cycle

Firstly, the pH and thermal co-response characteristics of AuNR@PNIPAM-VAA nanogel were preliminarily studied from the morphological and size characterization. Figure 2(a) is high-angle torus dark field (HAADF) image of AuNR@PNIPAM-VAA nanogel. The corresponding N-element mapping image which contributes from PNIPAM-VAA further proves that PNIPAM-VAA shell is successfully wrapped on AuNR. The combined images of Au and N elements show that AuNR@PNIPAM-VAA nanogel has a clear core-shell structure. Before TEM, DLS testing, the AuNR@PNIPAM-VAA nanogel was dispersed in PBS buffer at various pH values, then the solution was placed in a plastic tube and incubated in a water bath at the indicated temperature for 15 min. Then, the thermal response size and morphology changes of AuNR@PNIPAM-VAA nanogel in PBS buffers under the condition of collapse (50 ℃, pH = 5.8) and swelling (25 ℃, pH = 7.4) were characterized by TEM as shown in Fig. 2(b). In the swollen state (25 ℃, pH = 7.4), the diameter of PNIPAM-VAA shells was 220 nm. However, the diameter is about 150 nm in the state of collapse (50 ℃, pH = 5.8), and the volume of PNIPAM-VAA shells is reduced by about 68.30% after phase transformation. In addition, TEM images show that the single core-shell has a spherical shape and good dispersion. The hydrodynamic diameter and size distribution of AuNR@PNIPAM-VAA nanogel were measured by DLS technology. The results are shown in Fig. 2(c). The length of AuNR is about 130 nm, diameter is about 16 nm, and transverse diameter ratio is about 8, which is consistent with the TEM result. The hydrodynamic radius of AuNR@PNIPAM-VAA nanogel measured at collapse (50 ℃, pH = 5.8) and swelling (25 ℃, pH = 7.4) is about 406 nm and 452 nm, respectively. The changes tendency of hydrated particle size is consistent with TEM images, but the hydrated particle size is significantly larger than that of TEM results. This is because AuNR@PNIPAM-VAA nanogel in the measured hydrated particle size state contain a higher proportion of water. The results showed that the PNIPAM-VAA shell existed in a large swelling state in normal tissue at room temperature, and the swelling-collapse phase transition occurred with the increase of temperature in response to the low pH. Secondly, the variation of AuNR surface potential during the wrapping of PNIPAM-VAA shell was measured as shown in Fig. 2(d). The zeta potential of AuNR is about 39.48 mV, which decreases to 30.91 mV after VAA functionalization and − 21.97 mV after PNIPAM-VAA shell encapsulation, confirming the successful preparation of AuNR@PNIPAM-VAA core-shell structure.

In order to verify that AuNR@PNIPAM-VAA nanogel can only undergo thermal-sensitive phase transition at low pH in TME, the absorption spectra of AuNR@PNIPAM-VAA nanogel at temperature ranging from 34 ℃ to 46 ℃ were measured at pH 5.8 as simulated tumor extracellular matrix and at pH 7.4 as normal physiological environment, as shown in Fig. 2(e). The results show that the absorption spectrum of AuNR@PNIPAM-VAA nanogel remains unchanged before and after temperature change at pH 7.4. On the contrary, at pH 5.8, the longitudinal plasmon resonance absorption peak of AuNR@PNIPAM-VAA nanogel redshifts from 919 nm at 38 ℃ to 949 nm at 46 ℃ with increasing temperature in the range of 34 to 46 ℃. Figure 2(f) shows that the light absorption at 1064 nm of AuNR@PNIPAM-VAA nanogel at pH 5.8 is enhanced with increasing temperature. At the same time, quantitative calculation shows that the light absorption at 1064 nm was enhanced by 88.86% at pH 5.8. Figure 2(g) shows the relationship at pH 5.8 between temperature and light absorption of AuNR@PNIPAM-VAA nanogel at 1064 nm. VPTT is determined to be about 45 ℃ by calculating the first derivative of curve, and the enhancement rate of light absorption of AuNR@PNIPAM-VAA nanogel changes the fastest with temperature. To investigate the reversibility of thermal-sensitive phase transitions triggered by low pH of AuNR@PNIPAM-VAA nanogel, the light absorption changes at 1064 nm were measured at pH 5.8 during five consecutive heat-cooling cycles (from 30 °C to 60 °C) at temperatures above and below the midpoint of the VPTT. As shown in Fig. 2(h), periodic changes in light absorption are observed, demonstrating that AuNR@PNIPAM-VAA nanogel possesses the sustainability and repeatability of the phase transition, and the stability of the thermal-response. These results show that the thermal-sensitive phase transition of AuNR@PNIPAM-VAA nanogel is pH-dependent and highly reversible at low pH as in TME.

### Photothermal properties of AuNR@PNIPAM-VAA nanogel

**Fig. 3:**
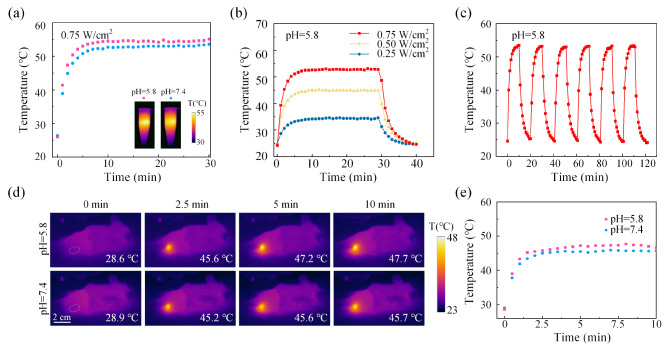
Photothermal conversion performance of AuNR@PNIPAM-VAA nanogel. (**a**) Temperature rise curve as a function of time; (**b**) Temperature changes under different power density laser irradiation; (**c**) Photothermal stability under cyclic irradiation; (**d**) Photothermal imaging and (**e**) real-time temperature rise curve of mouse subcutaneous tissues under irradiation after injection of AuNR@PNIPAM-VAA nanogel

The photothermal conversion of AuNR@PNIPAM-VAA nanogel in vitro was investigated due to the presence of AuNR nuclei with light absorption. As shown in Fig. 3(a), the temperature of the two groups AuNR@PNIPAM-VAA increased rapidly, and the temperature tended to equilibrium when the irradiation time reached 10 min. When the irradiation time reaches 30 min, the temperature of the group at pH 5.8 reaches 55.1 ℃, and the group at pH 7.4 is up to 53.6 ℃. Then, the temperature rise characteristics of AuNR@PNIPAM nanogel were measured under 980 nm CW laser irradiation with different optical power densities. Figure 3(b) shows that the higher laser power resulted in faster heating rate and higher final temperature. When the laser power reaches 0.75 W/cm^2^, the AuNR@PNIPAM-VAA solution temperature of 1 mg/mL AuNR@PNIPAM nanogel rapidly rises to about 52.5 ℃ in a short time (10 min). As shown in Fig. 3(c), the maximum temperature of AuNR@PNIPAM-VAA aqueous solution during six laser irradiation cycles (0.75 W/cm^2^, 10 min) is always about 53.3 ℃, and the peak shape has no significant change, indicating that these AuNR@PNIPAM-VAA nanogel has good photothermal stability. These results show that AuNR@PNIPAM-VAA nanogel has high efficiency and stable photothermal conversion, which is important for the in vivo application.

The photothermal effect of the AuNR@PNIPAM-VAA nanogel in vivo was monitored using an infrared thermal imager, showing a pH-dependent photothermal behavior. Two groups of AuNR@PNIPAM-VAA nanogel buffer (1 mg/mL) at pH 7.4 and 5.8 were injected into the subcutaneous tissue of normal mice respectively, and then real-time imaging was performed. Figure 3(d) records the photothermal images under irradiation with a laser power density of 0.5 W/cm^2^. As shown in the figure, the temperature of the simulated normal tissue area (pH = 7.4) in the control group increased rapidly with the extension of laser irradiation time. Meanwhile, the local temperature of the simulated tumor region (pH = 5.8) injected with the nanogel increased more significantly with irradiation time, while no significant temperature rise was observed in other body parts of the mice. Figure 3(e) statistically analyzes the changes of temperature rise in the area injected with AuNR@PNIPAM-VAA nanogel with irradiation time. Compared with the temperature rise in the simulated tumor area (pH = 5.8), the temperature rose rapidly to about 47.7 ℃, and the temperature in the simulated normal tissue area (pH = 7.4) finally stabilized at about 45.7 ℃. All of above results confirmed that AuNR@PNIPAM-VAA nanogel has excellent photothermal properties which can be dynamically modulated by laser irradiation time and power, and shows great potential to be the “switch” for transferring light to heat.

### Photoacoustic properties of AuNR@PNIPAM-VAA nanogel

**Fig. 4:**
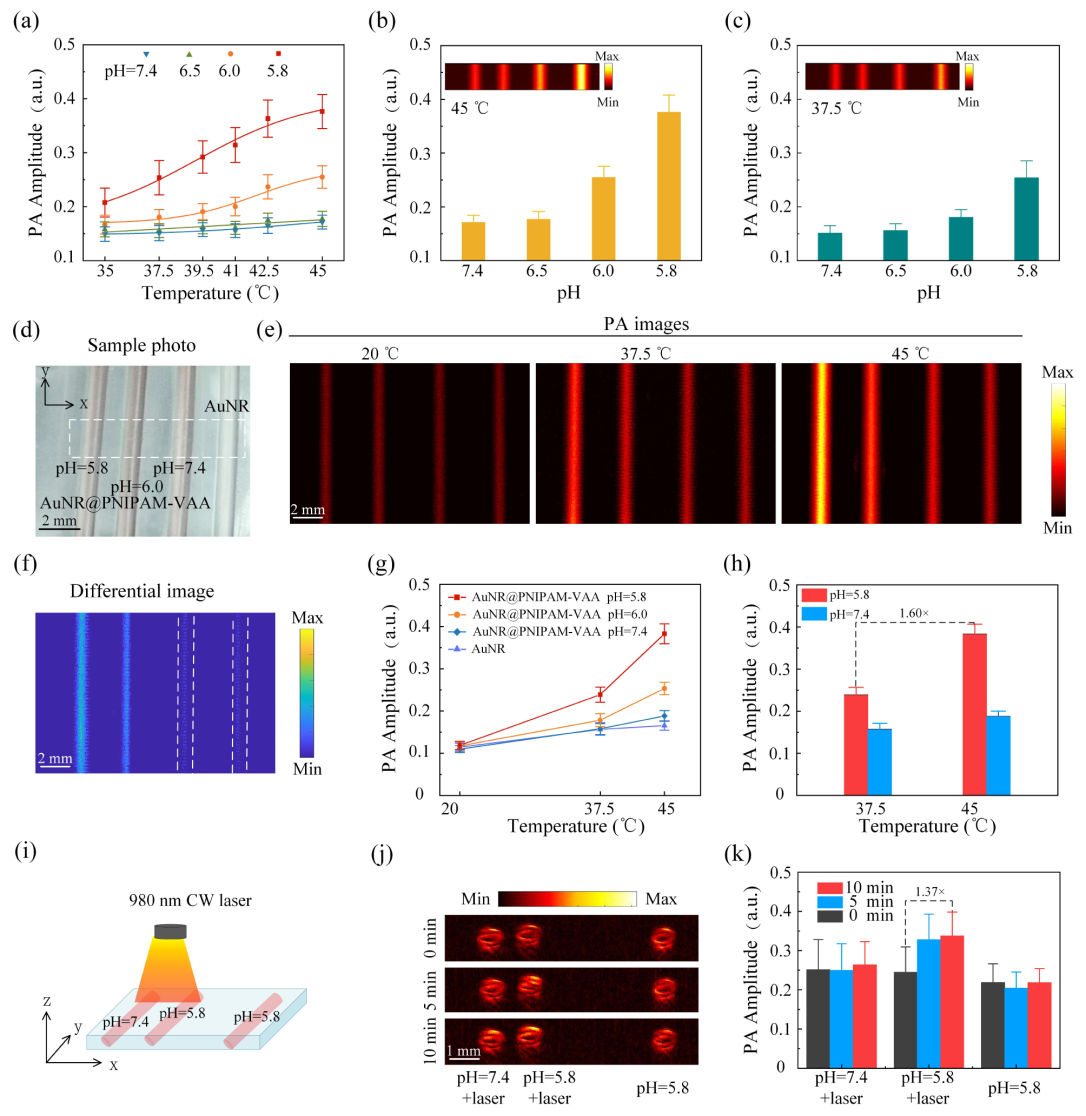
Photoacoustic properties of AuNR@PNIPAM-VAA nanogel. (**a**) Photoacoustic signal amplitudes; (**b**) and (**c**) Statistics signal amplitudes of AuNR@PNIPAM-VAA nanogel at different pH values at 45 ℃ and 37.5 ℃, respectively; (**d**) Photograph and (**e**) photoacoustic images in x-y plane of AuNR@PNIPAM-VAA nanogel and AuNR samples; (**f**) Differential photoacoustic images basing on the results at 45 °C and 37.5 °C in Fig. 4(e); (**g**) Statistic photoacoustic signal amplitude varied with temperature in Fig. 4(e); (**h**) Quantitative enhancement of photoacoustic signal; (**i**) Schematic diagram of AuNR@PNIPAM-VAA nanogel at pH 7.4 and pH 5.8; (**j**) Photoacoustic image in x-z plane at different exposure time; (**k**) Quantitative enhancement of photoacoustic signal after the irradiation of 980 nm CW laser (0.5 W/cm^2^)

Based on the above pH-triggered thermal property of AuNR@PNIPAM-VAA nanogel, Fig. 4(a) records the relationship between the photoacoustic signal amplitude at different pH and temperature. The results show that under neutral conditions (pH = 7.4), photoacoustic signal has hardly change with increasing temperature. Under acidic conditions (pH < 6), photoacoustic signal increases with increasing temperature; and with decreasing pH, the enhancement trend of photoacoustic signal becomes more and more significant. Figure 4(b) and 4(c) describe the statistical photoacoustic signal intensity of AuNR@PNIPAM-VAA nanogel with different pH value at 45 ℃ and 37.5 ℃, respectively. The insert were the corresponding images acquired by PAM at 1064 nm. The photoacoustic signal amplitude is inert to pH near the physiological temperature, and increases significantly with the decrease of pH near the hyperthermia temperature (45 ℃). In order to illustrate the synergistic effect of thermal and pH on PAI, AuNR@PNIPAM-VAA solution at different temperature and pH levels were imaged by a 1064 nm by PAM system. Figure 4(d) is the corresponding sample photo, and the white dotted line box indicates the imaging area. Point-by-point scanning in the x-y plane (C-scan) was performed, and the maximum value of the photoacoustic signal at each position is projected to obtain the photoacoustic image in the x-y plane as shown in Fig. 4(e). It can be clearly seen that the photoacoustic signal intensity of AuNR@PNIPAM-VAA nanogel increases with the increase of temperature under acidic conditions, but remains basically unchanged at pH 7.4. Figure 4(f) is the 45 °C and 37.5 °C differential image of Fig. 4(e), where AuNR@PNIPAM-VAA nanogel under acidic conditions are clearly identified owing to the enhancement of AuNR@PNIPAM-VAA photoacoustic signal under the synergistic effect of thermal and pH. The signal intensity of the sample in Fig. 4(e) was quantified and statistically analyzed to get Fig. 4(g). The results show that with the decrease of pH, the thermal induction enhancement effect of photoacoustic signal amplitude becomes more obvious. As shown in Fig. 4(h), when the sample is heated from 37.5 ℃ to 45 ℃, the photoacoustic signal amplitude is enhanced by 60% at pH 5.8 and slightly by 19% at pH 7.4. In summary, the photoacoustic signal of AuNR@PNIPAM-VAA nanogel shows a significant pH-dependent thermal-trigged enhancement in the pH range of 5.8 to 7.4, indicating that the nanogel can target tumor stroma at low pH.

To further verify that NIR light-switching stimulation (980 nm CW laser) can be used to regulate the thermal switch of the thermal-sensitive phase transition in the TME, PAI of simulated tissue samples on AuNR@PNIPAM-VAA nanogel were performed as shown in Fig. 4(i). Three polyethylene tubes were injected with 1 mg/mL AuNR@PNIPAM-VAA nanogel solution with different pH values. Two tubes with pH levels of 7.4 and 5.8 were irradiated with 980 nm CW laser (0.5 W/cm^2^) for 0, 5, and 10 min. Meanwhile, and the third tube containing AuNR@PNIPAM-VAA nanogel solution at pH 5.8 without light irradiation was used as a control. Point-by-point scanning in the x-axis direction (B-scan) and the photoacoustic signals in the z-axis direction at each position are projected to obtain x-z plane photoacoustic images at different exposure times were obtained as shown in Fig. 4(j). The photoacoustic signal of the nanogel solution with pH = 5.8 increased significantly with the increase of irradiation time under irradiation, however, the photoacoustic signal of the AuNR@PNIPAM-VAA nanogel buffer solution with pH 7.4 increased slightly. The photoacoustic signal intensity of the sample in Fig. 4(j) was quantified and statistically analyzed to obtain Fig. 4(k). The photoacoustic signal of AuNR@PNIPAM-VAA nanogel was enhanced by 37% in the simulated tumor region (pH = 5.8) after 10 min of laser irradiation, and the enhancement amplitude was only 5% in the simulated normal tissue region (pH = 7.4). The above results verified that the AuNR@PNIPAM-VAA nanogel has a pH-responsive photothermal conversion ability, and NIR light (980 nm) can be used to modulate the thermal field to trigger the thermal-sensitive phase transition.

### In vivo dynamic enhanced photoacoustic imaging of tumor

**Fig. 5:**
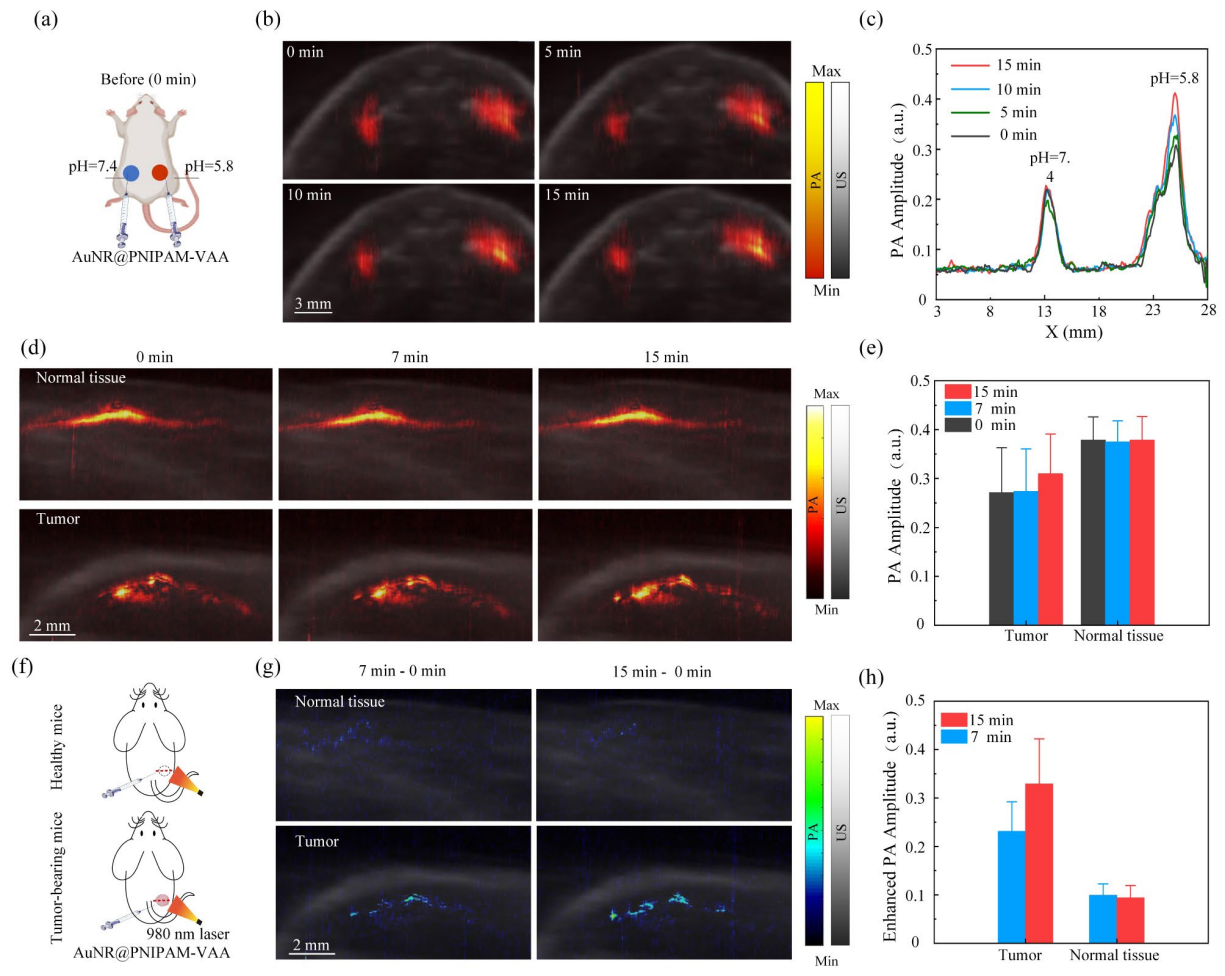
In vivo dynamic enhanced photoacoustic effect of the AuNR@PNIPAM-VAA nanogel for tumor imaging. (**a**) Schematic PAI of subcutaneous tissue after injection of AuNR@PNIPAM-VAA nanogel; (**b**) Superposition of photoacoustic and ultrasound images of subcutaneous tissues of normal mice injected with AuNR@PNIPAM-VAA nanogel; (**c**) Peak-to-peak projection curve of photoacoustic signal amplitude of subcutaneous tissue of normal mice in Fig. 5(b); (**d**) photoacoustic + ultrasound image of normal tissue and tumor; (**e**) Photoacoustic signal amplitude and (**f**) Schematic PAI of mice after injection of AuNR@PNIPAM-VAA nanogel; (**g**) Superposition of ultrasound image and differential photoacoustic image and (**h**) enhanced photoacoustic signals of normal tissue and tumor

To verify the possibility of AuNR@PNIPAM-VAA nanogel to distinguish the normal and tumor tissue, a mouse model simulating the TME was constructed and imaged, and the specific protocol is shown in Fig. 5(a). 1 mg/mL of AuNR@PNIPAM-VAA nanogel was prepared in PBS buffer that mimics TME (pH = 5.8) and normal physiology (pH = 7.4), respectively. The nanogel was injected into the subcutaneous tissue of the normal mice back, and irradiated by a 980 nm CW laser (0.5 W/cm^2^). The x-z plane point scan was performed on the area of interest to obtain the photoacoustic image in Fig. 5(b). The photoacoustic image intensity of the AuNR@PNIPAM-VAA nanogel in the simulated tumor region (pH = 5.8) gradually increased with the increase of irradiation time, while the photoacoustic image intensity in the simulated normal tissue region (pH = 7.4) remained almost constant. In order to quantitatively compare the differences, we depicted the maximum projection curve of photoacoustic signal under different 980 nm laser irradiation time as shown in Fig. 5(c). The results showed that the photoacoustic signal in the simulated tumor area was enhanced by 35.5% after 15 min of exposure. The above results demonstrated the possibility that the prepared AuNR@PNIPAM-VAA nanogel can achieve tumor-specific high-contrast imaging by utilizing a thermal-sensitive phase transition triggered in response to the low pH in TME.

Photoacoustic + ultrasound image of normal tissue and tumor was presented in Fig. 5(d). Figure 5(e) shows the statistical analysis of the photoacoustic signal amplitude after different laser irradiation time. Results show that photoacoustic signal in the tumor region increased after laser irradiation, while that in the normal tissue presents limited differences. All of these can be ascribed to the pH dependence of AuNR@PNIPAM-VAA nanogel. Point-by-point photoacoustic scanning along the dashed line was shown in Fig. 5(f). Fig.  5(g) shows the superposition of ultrasound image and differential photoacoustic image of normal tissues and tumors after injection of AuNR@PNIPAM-VAA nanogel. The results showed that the enhanced photoacoustic intensity in the tumor region increased significantly with the increase of irradiation time, while that of the normal tissue remained almost unchanged. Figure 5(h) shows the quantified statistical analysis of the enhanced photoacoustic signal. After exposed to 980 nm CW laser with 15 min (0.5 W/cm^2^), the enhanced photoacoustic amplitude increased by 42% compared to that with 7 min laser irradiation in the tumor region. However, the photoacoustic amplitude of the subcutaneously injected AuNR@PNIPAM-VAA nanogel in the control group has slightly change. Thus, AuNR@PNIPAM-VAA exhibits strong enhanced photoacoustic properties in acidic TME based on its thermal-triggered NIR-II light absorption enhancement. These results indicate that the prepared AuNR@PNIPAM-VAA nanogel can achieve tumor-specific high-contrast PAI by dynamically adjusting the laser irradiation.

### In vivo toxicity assessment

**Fig. 6:**
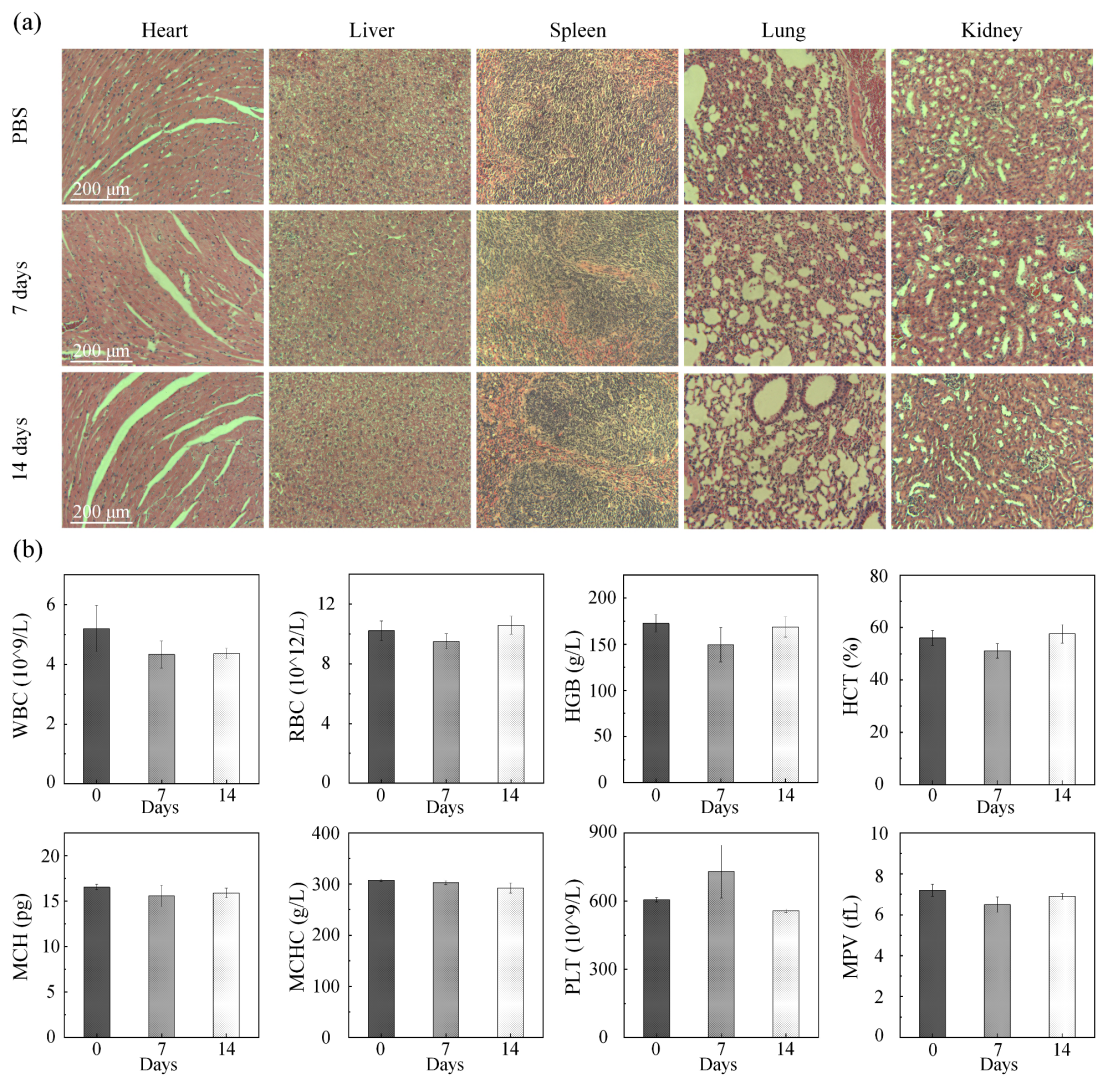
(**a**) H&E staining of different organs, (**b**) Serum biochemical and hematological analysis of healthy mice after injection of PBS and AuNR@PNIPAM-VAA nanogel for 7 days and 14 days

The pathological structure of the major organs of mice injected with PBS and AuNR@PNIPAM-VAA nanogel for 7 and 14 days was evaluated as shown in Fig. 6(a). The AuNR@PNIPAM-VAA nanogel did not damage these major organs, such as heart, liver, spleen, lung and kidney. This was followed by intravenous injection of AuNR@PNIPAM-VAA nanogel in three mice, and then serum biochemical and hematological parameters were systematically studied at 7 and 14 days. The results in Fig. 6(b) showed that there were no obvious abnormal changes in the test group compared with the PBS group, indicating that these AuNR@PNIPAM-VAA nanogels did not cause obvious infection and inflammatory response in the mouse model. The above results indicated that the toxic side effects of AuNR@PNIPAM-VAA nanogel were negligible.

## Conclusions

In conclusion, a volume phase-transition photoacoustic nanogel AuNR@PNIPAM-VAA has been successfully fabricated for tumor-specific high-contrast imaging. VAA monomer was introduced into PNIPAM chain to make the nanogel with pH responsive function. By adjusting the proportion of VAA monomers, PNIPAM-VAA shell obtained the pH-dependence required for thermal-sensitive volume phase transitions to target acid TME. As the temperature exceeded VPTT, the developed AuNR@PNIPAM-VAA nanogel underwent a solgel phase transition in TME and further resulted in enhanced NIR-II light absorption due to the change of nanogel’ refractive index. Based on the pH-dependence of PNIPAM-VAA thermal-sensitive phase transition, AuNR@PNIPAM-VAA nanogel can target TME at low pH and exhibit strong and switchable NIR-II light absorption under NIR light stimulation (980 nm), thereof AuNR@PNIPAM-VAA nanogel in the tumor region exhibited enhanced photoacoustic signal in the NIR-II window. In addition, the nanogel showed good biocompatibility, and no side effects were observed both in vitro and in vivo tests. However, it should be noted that the imaging wavelength is not the absorption peak of AuNR@PNIPAM-VAA, in which the nanoprobe produces the maximum photoacoustic efficiency. In further studies, we commit to develop new materials with strong absorption in the NIR-II region to achieve deeper and more sensitive PAI. In a word, a method of tumor-specific PAI using NIR light to regulate the thermal field and target the low pH TME is proposed, which is expected to realize accurate and dynamic monitoring of tumor diagnosis and treatment.

## Data Availability

The data that support the findings of this study are available from the corresponding author upon reasonable request.

## Abbreviations

NIR: II Second Near-Infrared
AuNR: Gold Nanorod
PNIPAM: Poly(n-isopropylacrylamide)
VAA: Vinyl Acetic Acid
TME: Tumor Microenvironment
PAI: Photoacoustic Imaging
PAM: Photoacoustic Microscopy
VPTT: Volume Phase Transition Temperature
